# Evidence of Field-Evolved Resistance of *Spodoptera frugiperda* to Bt Corn Expressing Cry1F in Brazil That Is Still Sensitive to Modified Bt Toxins

**DOI:** 10.1371/journal.pone.0119544

**Published:** 2015-04-01

**Authors:** Rose Monnerat, Erica Martins, Cristina Macedo, Paulo Queiroz, Lilian Praça, Carlos Marcelo Soares, Helio Moreira, Isabella Grisi, Joseane Silva, Mario Soberon, Alejandra Bravo

**Affiliations:** 1 Embrapa Recursos Genéticos e Biotecnologia, Brasília, Distrito Federal, Brazil; 2 Instituto Mato-Grossense do Algodão, Cuiabá, Mato Grosso, Brazil; 3 Departamento de Microbiologia, Universidade de Brasília, Brasília, Distrito Federal, Brazil; 4 Instituto de Biotecnologia, Universidad Nacional Autonoma de México, Cuernavaca, Morellos, Mexico; University of Tennessee, UNITED STATES

## Abstract

Brazil ranked second only to the United States in hectares planted to genetically modified crops in 2013. Recently corn producers in the Cerrado region reported that the control of *Spodoptera frugiperda* with Bt corn expressing Cry1Fa has decreased, forcing them to use chemicals to reduce the damage caused by this insect pest. A colony of *S*. *frugiperda* was established from individuals collected in 2013 from Cry1Fa corn plants (SfBt) in Brazil and shown to have at least more than ten-fold higher resistance levels compared with a susceptible colony (Sflab). Laboratory assays on corn leaves showed that in contrast to SfLab population, the SfBt larvae were able to survive by feeding on Cry1Fa corn leaves. The SfBt population was maintained without selection for eight generations and shown to maintain high levels of resistance to Cry1Fa toxin. SfBt showed higher cross-resistance to Cry1Aa than to Cry1Ab or Cry1Ac toxins. As previously reported, Cry1A toxins competed the binding of Cry1Fa to brush border membrane vesicles (BBMV) from SfLab insects, explaining cross-resistance to Cry1A toxins. In contrast Cry2A toxins did not compete Cry1Fa binding to SfLab-BBMV and no cross-resistance to Cry2A was observed, although Cry2A toxins show low toxicity to *S*. *frugiperda*. Bioassays with Cry1AbMod and Cry1AcMod show that they are highly active against both the SfLab and the SfBt populations. The bioassay data reported here show that insects collected from Cry1Fa corn in the Cerrado region were resistant to Cry1Fa suggesting that resistance contributed to field failures of Cry1Fa corn to control *S*. *frugiperda*.

## Introduction

Genetically modified (GM) plants expressing insecticidal proteins from the bacterium *Bacillus thuringiensis* (Bt) have been used in the field since 1996 and are important tools to control insect pests reducing the use of chemical insecticides [1]. In Brazil, the first commercial release of a transgenic crop for insect control was Bt cotton, occurring in 2005. The Bt Cry toxins are highly specific proteins that kill insects and are believed to be biodegradable. Bt bacteria produce several classes of Cry toxins [2] but those from Cry three-domain Cry family (3d-Cry) represent the most studied and the most used proteins in diverse GM crops. These toxins kill insect pests because they specifically interact with certain receptors found in the insect gut and induce the formation of pores in the apical membrane of the cells, destroying the intestinal tissue allowing bacterial septicemia in the hemocoel and resulting in larval death [3, 4].

In 2013, the global area planted with GM Bt plants was 75.9 million ha in different countries [5]. The area cultivated with GM soybean, corn and cotton in 2013 in Brazil was 40.3 million ha, placing it as the second largest country to grow GM plants in the world [5]. In 2007 several corn events expressing Bt toxins were commercially released in Brazil. These GM plants express one to three Bt proteins such as Cry1Ab, Cry1Ac, Cry1A.105, Cry1Fa, Cry2Ab2, Cry3Bb1 and Cry35Ab1 proteins [6]. In 2010/11 GM corn accounted for 57% of the total area of cultivated corn in Brazil [7]. The main grain and fiber producing regions of the country where GM plants are grown are the Midwest (Cerrado region), South and Southeast regions. The Cerrado is a savanna-type biome with a surface of two million km^2^, constituting one of the largest areas of agricultural activity in Brazil, as it represents almost 25% of the national territory. Annual cultivation of corn, soybean and cotton in the Cerrado begins in October and extends until June [8]. This area is planted with predominately Bt or herbicide-tolerant corn. Also, GM cultivars of soybean and cotton resistant to insects and herbicides are grown in this area.

Insects can evolve resistance to Bt toxins in laboratory and field conditions [1, 9, 10]. At least eight different insects species all over the world have already evolved resistance to Bt toxins in field conditions or in green houses [1, 11–18], representing the primary threat to the long-term efficacy of Bt toxins. One of the established strategies to delay the evolution of insect resistance to Bt-crops is the use of refuge areas that do not contain Bt plants. Refuges allowed the survival of susceptible insects that could mate with the few resistant insects selected in Bt-crops. If initial frequency of the resistance allele is low and is conferred by a recessive trait, the heterozygous progeny of this cross would present high susceptibility to Cry toxin expressed in Bt-crops. It is crucial that the dose of Bt toxin expressed in the Bt-plant is sufficient to kill heterozygous insects. This strategy is effective in delaying the evolution of insect resistance to Bt-crops [10].

The area of refuge recommended for the cultivation of GM corn in Brazil, was equivalent to 10% of the total area planted with Bt corn and that it should be located at a distance less than 800 meters from the Bt corn field [12]. Furthermore, the use of an insulation area of 100 meters surrounding Bt corn fields was established to avoid potential contamination of native cultures with GM crops. These recommendations, however, are not mandatory [7, 12]. In 2013 it was reported that areas of refuges were being implemented below the recommended level or were not used at all [12].

Resistance of *S*. *frugiperda* populations to Cry1Fa Bt corn was previously reported in Puerto Rico [13]. From 2010–2014, farmers of the Brazilian Cerrado region reported that the control effectiveness of Cry1Fa Bt corn decreased, forcing them to use chemical insecticides to reduce the damage caused by *S*. *frugiperda* [12], suggesting that insect resistance to Cry toxins might have evolved also in this region. High resistance levels of *S*. *frugiperda* populations to Cry1Fa corn in different regions of Brazil including state of Goiás in the Cerrado region was confirmed [14]. The resistance phenotype in these populations was conferred by one or more recessive alleles at the same locus and the frequency of resistant alleles increased significantly in the field during the past years [14].

Field-evolved resistance in different insect pests in other regions such as *Pectinophora gossypiella* on Cry1Ac Bt cotton in India [15], *Busseola fusca* on Cry1Ab Bt corn in South Africa [16], *Helicoverpa zea* on Cry1Ac Bt cotton in USA [1], *Diabrotica virgifera* in Cry3Bb Bt corn in USA [17] and *Helicoverpa armigera* on Cry1Ac Bt cotton in China [18] were also reported.

The objective of this study was to further analyze the *S*. *frugiperda* population that has evolved resistance to Cry1Fa toxin resulting in the survival of this insect pest in Cry1Fa Bt corn. We analyzed the stability of the resistant phenotype after several generations without selection pressure, studied the cross-resistance of this population to other Cry1A and Cry2A toxins and determined whether Cry1AMod toxins that have been reported as effective for the control of various lepidopteran species resistant to Cry1A toxins [19, 20] could be effective in controlling these *S*. *frugiperda* larvae collected from Brazilian Bt corn fields.

## Materials and Methods

### Location for insect collection

Insects were collected during 2013 on a farm in the municipality of Cabeceiras in the state of Goiás, located in the Cerrado region of West-Central Brazil (Latitude: -15.7961, Longitude: -46.927 15° 47 &rsquo;46 "South, 46° 55' 37 "West). The property is about 700 acres where Bt corn, conventional corn and herbicide tolerant soybeans and common beans are grown. Cry1Fa Bt corn had been cultivated for four years and Cry1A.105 corn, Cry2Ab corn and herbicide tolerance corn had been cultivated for the past two years. Insect collection activity and maintenance was authorized by the Ministry of Environment of Brazil (MMA), Instituto Chico Mendes de Biodiversidade—ICMBio, Sistema de Autorização e Informação em Biodiversidade-SISBIO Number: 39022–1.

### Establishment of an insect colony from survival insects collected on Bt corn fields

Four hundred *S*. *frugiperda* larvae were collected in 2013 from Cry1Fa Bt corn variety 30F53H, taken to the insect breeding laboratory at Embrapa Genetic Resources and Biotechnology and maintained individually until pupating in 50 ml plastic cups with acrylic covers containing 10 ml artificial diet [21]. The pupae were placed in cages internally wrapped in paper for oviposition with 10% honey for emerging adults feeding. Egg masses were collected, surface-sterilized in 10% Na hypochlorite for two min and placed in new plastic cups containing artificial diet. Upon reaching the third larval stage, the larvae were separated to avoid cannibalism and incubated to reach the pupal stage, after which the procedure described above was repeated. The rearing temperature was 28 ± 2°C, relative humidity RH: 60%, and photoperiod of 14:10 (light: dark). This colony is being reared on artificial diet without added toxin, i.e., without selection pressure, and named SfBt.

The susceptible colony of *S*. *frugiperda* originated from mass rearing in Embrapa, and named SfLab. This breeding was implemented in 1988 [21] and the insects have never been exposed to Bt toxins.

### Bioassays

Bioassays were performed with activated Cry1Fa toxin (purified and kindly supplied by Dr. Marianne Carey from Case Western Reserve University-School of Medicine, Cleveland, OH) and a mixture of spores and crystals from Cry1Aa, Cry1Ab, Cry1Ac, Cry2Aa, Cry2Ab, Cry1AbMod and Cry1AcMod produced in Bt transformant strains. The *cry1Aa*, *cry1Ab*, *cry1Ac*, *cry2Aa*, *cry2Ab* genes, obtained from HD-1 strain were individually cloned into pHT315 vector [22] and expressed in *B*. *thuringiensis* serovar. *israelensis* acrystalliferous strain 4Q7 (*Bacillus* Genetic Stock Center) [23]. The Cry1AbMod and Cry1AcMod proteins were also constructed using pHT315 vector and expressed in Bt as described previously [19]. The recombinant strains were grown in a rotary incubator at 200 rpm for 72 hours in EMBRAPA medium [24] supplemented with 10 μg/ml of erythromycin. After this period, 95% sporulation of bacteria was confirmed by observation through a phase contrast microscope. The cultures were centrifuged at 12,000 *xg* for 30 min at 4° C. The spore-crystal mixtures were frozen and lyophilized for 18 h in a Labconco Lyphlock model lyophilizer. Crystal protein concentration was measured by using the Bradford reagent from BioRad. Bioassays were carried out by spreading 35 μl of 10 serial dilutions containing different protein concentrations of Cry1Fa protein or mixture of freeze-dried spore crystals, on the surface of artificial diet previously distributed in 24-well plates. After the protein samples were dried in the diet, one second-instar larvae was placed in each well. We tested 24 larvae per toxin concentration in triplicate, and analyzed ten different concentrations of toxin, making a total of 720 larvae per LC_50_ value determination. As negative control one plate was left without toxin addition. Three biological replicates were performed. The first reading of mortality was made 48 hours after start of the test, at which time the larvae were transferred to 50 ml plastic cups containing diet without toxin. On the seventh day, the second and final reading was taken [25, 26]. The analysis of mortality data, obtained after 7 days were accomplished in Team R program [27], where the LC_50_ was determined using generalized linear models (GLM) with probit link function (Probit analysis)[28]. The confidence intervals of 95% for the LC_50_ and the slope of the dose were calculated. This assay is accredited by ISO/IEC 17025. Bioassays were performed with larvae of the F1 generation of SfBt and SfLab during 2013. Bioassays were also performed with larvae from F2 and F8 generations of SfBt colony in 2013 and 2014 respectively, using a diagnostic dose corresponding to 10 times the LC_50_ dose on the SfLab strain (3,500 ng/cm^2^). Bioassays for other Cry and Cry1AMod toxins were performed as described above with larvae from the F8 generation.

Bioassays with corn leaves were also analyzed under laboratory conditions. Corn leaves from Cry1Fa corn variety 30F53H were collected during 2013 in the Cabeceiras Cerrado region of Brazil. Leaves from non transgenic plants variety 30F53 were also collected from the same region at the same time. Leaves were taken to the insect breeding laboratory at Embrapa Genetic Resources and Biotechnology and maintained at-20°C. Frozen leaves were defrost in February 2014 and placed in Petri dishes containing a moisture paper with water. A total of 20 larvae in the second instar from SfLab and SfBt (8^th^ generation) were used per treatment in three repetitions. Mortality was recorded after 7 days.

### Binding assay of recombinant Cry proteins to BBMV isolated from *S*. *frugiperda*

The mixture of spores/crystals produced by the recombinant Bt strains was washed three times with PBS buffer (150 mM NaCl, 2.8 mM NaH_2_PO_4_, 4 mM Na_2_HPO_4_.7 H_2_O, pH 7.2) with 1 mM EDTA and 0.1 mM PMSF. The crystals were separated by sucrose gradient centrifugation [29] and solubilized in alkaline buffer (50 mM Na_2_CO_3_, 0.2% β-mercaptoethanol, pH 10.5). The protoxins were activated with trypsin (1:50 w/w) (TPCK, Sigma Aldrich, St. Louis, MO) for 2h at 37° C; the reaction was stopped by addition of PMSF (1 mM final concentration). Activated toxins were purified as previously described [3]. The protein concentration was determined using Bradford reagent from BioRad. The integrity and relative purity of the 130 kDa protoxins and 60 kDa activated toxins was analyzed by 12% SDS- PAGE.

Brush border membrane vesicles (BBMV) from the larval gut were purified according to Wolfersberger *et al*. [30], from midgut tissue of third instar larvae of SfBt (F8 generation) and SfLab populations in February 2014. Briefly the midgut tissues of the larvae were removed with forceps, washed and stored in buffer (200 mM mannitol, 1 mM DTT and 1 mM HEPES-Tris, pH 7.4) at-80°C. One gram of midgut tissue was homogenized in 10 ml of solution I (300 mM Mannitol, 17 mM Tris-HCl, 10 mM HEPES, 5 mM EGTA, 2 mM DTT, 1 mM EDTA, 1 mM PMSF, pH 7 4), nine strokes were given at 2,250 rpm in a Polytron homogenizer-blender. Then, 10 ml of cold 24 mM MgCl_2_ was added and the contents were mixed gently and incubated on ice for 15 min. The reaction was centrifuged at 2,500 *xg* for 15 min at 4° C. The supernatant was transferred to another tube and centrifuged again at 30,000 *xg* for 30 min at 4° C. The supernatant was discarded and the pellet suspended in 5 ml of solution I and 5 ml of 24 mM MgCl_2_ were added, mixed gently and incubated on ice for 15 min. The reaction was centrifuged at 2,500 *xg* for 15 min at 4° C. The supernatant was transferred to another tube and centrifuged again at 30,000 *xg* for 30 min at 4° C. The supernatant was again discarded and the pellet suspended in 5 ml of Milli-Q cold water. Three additional strokes at 2,250 rpm were given in the homogenizer device. Finally, the sample was dialyzed once against one l of buffer (150 mM KCl, 2 mM EGTA, 0.5 mM EDTA, 10 mM HEPES pH 7.4, 4° C), aliquoted and stored at-80° C. Enrichment of 4–6 fold of ALP enzymatic activity compared to the initial gut homogenate was observed in BBMV preparations.

### Biotinylation of proteins

The trypsin activated proteins Cry1Aa, Cry1Ab, Cry1Ac, Cry2Aa, Cry2Ab and Cry1Fa were biotinylated using the ECL Protein Biotinylation System (GE Healthcare) kit according to the manufacturer's recommendations. After biotinylation, detection was performed with peroxidase-conjugated streptavidin to confirm the biotin labeling of proteins as previously reported [31] using streptavidin-peroxidase conjugate (1:6000-GE Healthcare) for 1h at 25°C, followed by incubation with SuperSignal chemiluminescence substrate (Pierce) according to the manufacturer's recommendations.

### Binding assay of biotin-labeled Cry toxins to BBMV membranes from susceptible and Cry1Fa resistant *S*. *frugiperda* populations

Binding analyses were performed in February 2014 using BBMV from SfBt and SfLab populations as previously reported [31]. Briefly, 10 μg of *S*. *frugiperda* BBMV were incubated with10 nM of each biotinylated toxin for 30 min at 25°C in 100 μl total volume of binding buffer (0.1% PBS, 0.1% BSA, 0.1% Tween 20, pH 10.5). Unbound toxins were removed by centrifugation (10 min at 14,000 *xg*). The BBMV containing bound Cry toxins were washed three times in binding buffer and suspended in 15 μl of 1X PBS and 5 μl of 4X Laemmli sample buffer (0.125 M Tris/HCl, 4% SDS, 20% glycerol, 10% 2-mercaptoethanol, 0.01% Bromophenol Blue, pH 6.8). The samples were boiled for 3 min, resolved by SDS-PAGE at 9% and transferred to nitrocellulose membrane as described above. Labeled proteins bound to BBMV were visualized with streptavidin-peroxidase conjugate followed by incubation with SuperSignal chemiluminescence substrate as described above. The optical density of the bands was scanned with ImageJ program and the percentage of each band on the blot was calculated.

## Results

### Susceptibility of *S*. *frugiperda* populations to Cry1Fa

The Cry1Fa lethal concentration required to kill 50% of *S*. *frugiperda* larvae (LC_50_) from the different populations was determined. Table 1 shows that the LC_50_ value was at least ten times greater in the SfBt population compared with the control population SfLab. The exact LC_50_ values could not be calculated for the SfBt colony since the highest dose of Cry1Fa that we could assayed was 3500 ng/cm^2^ and at this concentration the mortality was not enough to determine LC_50_ values.

**Table 1:** Toxicity of different Cry toxins to *S*. *frugiperda* colonies of SfLab and SfBt.

	**SfLab**			**SfBt**			
**Protein**	**LC**_50_ ^a^ **ng/cm** ^2^	**Slope**	**Standard error**	**LC**_50_ ^a^ **ng/cm** ^2^	**Slope**	**Standard error**	**RR**
Cry1Fa	342 (192–609)	1.3	1.0	> 3,500	-	-	> 10
Cry1Aa	73.0 (26–120)	0.0061	0.0020	2514 (1283–3745)	0.00048	0.00017	34
Cry1Ab	403 (251–647)	1.8	1.1	1636 (843–2429)	0.0010	0.00030	4.0
Cry1Ac	545 (318–771)	0.0010	0.00030	1415 (789–2040)	0.00090	0.00030	2.5
Cry2Aa	668 (424–912)	0.0020	0.00050	827 (562–1093)	0.0020	0.0010	1.2
Cry2Ab	> 3,500	-	-	> 3,500	-	-	1.0
Cry1AbMod	60 (42–86)	1.8	1.1	191 (112–326)	1.5	1.1	3.1
Cry1AcMod	44 (29–68)	1.7	1.1	249 (134–462)	1.5	1.1	5.6

^a^ LC_50_ analyzed by Probit program R Core Team Software (fiducial level 95%). RR. Resistance ratio SfBt LC_50_/LC_50_ SfLab.

The colony SfBt was maintained in the laboratory in the absence of selection pressure for eight generations. Each of these generations was used to determine their susceptibility to a Cry1Fa diagnostic concentration (3,500 ng/cm^2^) 10 times higher than the LC_50_ obtained with Cry1Fa in larvae of the SfLab colony (Table 1). At this concentration the mortality of SfLab was 100% and mortality of F1 of SfBt was less than 5%. Mortality of insects obtained with F2 and F8 generations of SfBt was also less than 5% and toxin susceptibility was not significant different, suggesting that in this population the character of resistance was already established in the field when we isolated the first generation of insects.

In order to determine whether the level of resistance of the SfBt population to Cry1Fa affects the efficacy of Cry1Fa corn plants, the number of survival larvae of the susceptible SfLab and the Cry1Fa-resistant population SfBt on leaves of Bt corn plants was determined. The larvae of the population SfLab did not survive on leaves from Cry1Fa corn while the F8 generation of SfBt population showed 100% survival. The SfLab and SfBt populations had similar 100% survival on leaves from non-transgenic corn.

### Susceptibility of *S*. *frugiperda* populations to other Cry protoxins

Subsequently, we compared the susceptibility of the two *S*. *frugiperda* populations to other Cry proteins as described in Materials and Methods. The F8 generation of SfBt colony showed LC_50_ values 30 times higher for Cry1Aa toxin than those of the susceptible SfLab colony (Table 1). The LC_50_ values of Cry1Ab and Cry1Ac were 4 and 2.5-fold higher respectively when compared to the values obtained in the SfLab colony. The fiducial limits values for these bioassays do not overlap, indicating that these differences are significant. In contrast, analysis of susceptibility to Cry2Aa and Cry2Ab showed that there are not significant differences in susceptibility to these toxins which showed low or no-toxicity to both SfLab and SfBt insects (Table 1). These data show that the Cry1Fa resistant population SfBt shows higher cross-resistance to Cry1Aa toxin and low cross-resistance to Cry1Ab and Cry1Ac. No cross-resistance to Cry2A toxins was observed, although Cry2A toxins would not be an alternative to control *S*. *frugiperda* larvae in the Cerrado region of Brazil due to their low toxicity. It is worth to mention that these assays were performed with spore/crystal suspensions. In all cases the same acrystalliferous Bt 4Q7 strain was transformed with the pHT315 vector containing the different *cry* genes as reported in Material and Methods section. There is evidence in the literature that Bt spores produce virulence factors that could be important for toxicity [32]. However, since the same Bt spores are present in these bioassays and no toxicity was observed in the bioassays performed with the Cry2Ab transformat strain, we could conclude that the effect of spores in toxicity to these populations is not relevant.

Toxicity assays of Cry1AbMod and Cry1AcMod toxins showed that these toxins are highly active against both the SfLab and the SfBt populations. The Cry1AMod proteins showed an important insecticidal activity against the SfBt population. In the case of the SfLab population Cry1AbMod and Cry1AcMod showed insecticidal effect comparable to that of Cry1Aa (Table 1). However, Table 1 also shows that Cry1AbMod and Cry1AcMod toxins have cross-resistance ratios of 3.1 and 5.6, respectively. Although SfBt larvae show cross-resistance to Cry1AMod toxins, the potency ratio of Cry1A/Cry1AMod, calculated as the LC_50_ value of a native toxin divided by the LC_50_ of the corresponding modified toxin, is very high in the resistant strain since Cry1AbMod show a potency of 8.5 fold higher than the native Cry1Ab toxin and Cry1AcMod of 5.7 fold higher than Cry1Ac against the SfBt population (Table 2). It would be interesting to calculate the potency of Cry1AMod compared to Cry1Fa (Cry1Fa/Cry1AMod). However, we cannot directly compare the potency of these proteins since bioassays of Cry1Fa were performed with purified protein and those of Cry1AbMod or Cry1AcMod were done with a mixture of spore/crystals. Even though that we can not give an exact value of potency of Cry1Fa/Cry1AMod, the data of the LC_50_ value of a Cry1Fa toxin and of the modified toxins against SfBt (Table 1) indicate that Cry1AMod toxins show higher insecticidal effect than Cry1Fa against SfBt strain. These data indicate that Cry1AMod toxins could be an alternative to control SfBt larvae.

**Table 2:** Potency ratio for Cry1AMod toxin relative to native toxins.

Toxin pair	SfLab	SfBt
Cry1Ab /Cry1AbMod	6.7	8.5
Cry1Ac/Cry1AcMod	12	5.7

LC_50_ of a native toxin divided by the LC_50_ of a modified toxin (based on data from Table 1). Potency ratios > 1 indicates the modified toxin was more potent than the native toxin

### Binding assays of Cry toxins to *S*. *frugiperda* brush border membranes

**Fig 1:**
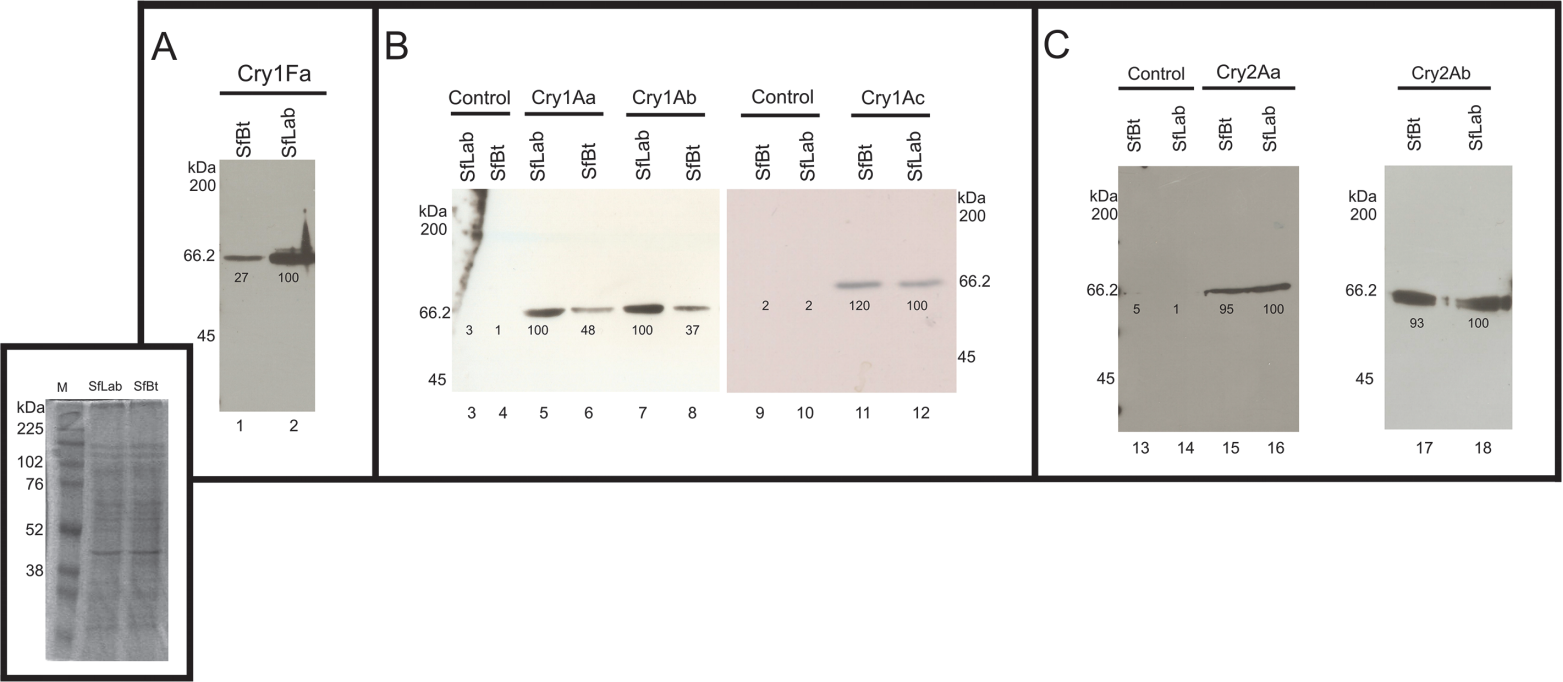
Analysis of binding of Cry toxins to BBMV from SfLab and SfBt populations. Panel A, Binding of Cry1Fa: Lane 1, binding of Cry1Fa to SfBt; Lane 2, binding of Cry1Fa to SfLab: **Panel B,** Analysis of binding of Cry1A toxins: Lanes 3 and 10, control of BBMV from SfLab without toxin incubation; Lanes 4 and 9, control of BBMV from SfBt without toxin incubation; Lane 5, binding of Cry1Aa to SfLab; Lane 6, binding of Cry1Aa to SfBt; Lane 7, binding of Cry1Ab to SfLab; Lane 8, binding of Cry1Ab to SfBt; Lane 11, binding of Cry1Ac to SfBt; Lane 12, binding of Cry1Ac to SfLab. Panel C, binding of Cry2A toxins: Lane 13, control of BBMV from SfBt without toxin incubation; Lane 14, control of BBMV from SfLab without toxin incubation; Lane 15 binding of Cry2Aa to SfBt; Lane 16, binding of Cry2Aa to SfLab; Lane 17, binding of Cry2Ab to SfBt; Lane 18, binding of Cry2Ab to SfLab. Binding of biotinylated toxins (10 nM) to BBMVs from *S*. *frugiperda* SfLab and SfBt populations was performed as described in material and methods. Numbers under the bands represent the percentage of each band on the blot calculated after scanning optical density of the bands with ImageJ program and using one band of similar size in the gel as 100% reference. Insert: Gel 12% SDS PAGE with BBMVs prepared from SfLab and SfBt populations. Lane 1, Rainbow molecular marker (GE); Lane 2, 10 μg of BBMV SfLab; Lane 3, 10 μg of BBMV SfBt

Previously it was shown that binding of Cry1A and Cry1Fa toxins to *S*. *frugiperda* BBMV is specific [33]. We analyzed the binding of different Cry toxins to BBMV from both *S*. *frugiperda* populations. Fig. 1A shows that Cry1Fa toxin is able to bind to BBMV prepared from SfBt and SfLab strains, but showed a significant greater binding to SfLab BBMV compared to BBMV from SfBt. Densitometry of the bands indicated that Cry1Fa binds at least 73% less efficiently to BBMV from SfBt compared to BBMV from SfLab. Fig. 1B shows that the three Cry1A toxins were able to bind to membranes of both insect populations. Also, that Cry1Aa and Cry1Ab bound better to BBMV from SfLab than to BBMV from SfBt. Densitometry of the bands indicated that these toxins bind with 57% and 63% less efficiency to the BBMV from SfBt, respectively. Fig. 1C shows that Cry2A toxins also bound to BBMV from SfLab and SfBt but only 5–7% difference in binding was observed between both insect populations.

Homologous and heterologous binding competition analysis of Cry1Fa toxin to *S*. *frugiperda* BBMV showed that Cry1Fa share binding sites with Cry1A toxins but not with Cry2A [33]. We performed homologous and heterologous competition binding assays of biotin-labeled Cry1Fa to SfLab BBMV confirming that Cry1Fa binding is specific and that Cry1Aa, Cry1Ab or Cry1Ac toxins efficiently compete the binding of labeled Cry1Fa to BBMV. In contrast, Cry2Aa and Cry2Ab did not compete Cry1Fa binding to BBMV (data not shown).

## Discussion

In this work we report the establishment of a *S*. *frugiperda* colony with significant resistance to Cry1Fa toxin from individuals collected in a region where Bt corn expressing the Cry1Fa toxin was grown for four years. Recently, a parallel study also reported high resistance levels of *S*. *frugiperda* populations to Cry1Fa corn in the same Brazilian region [14]. Our data regarding the characterization of the resistant population SfBt from de Cerrado region shows that the resistant allele responsible for resistance is stable in the population since after eight generations without selection pressure, the resistance ratio remained high.

The resistance mechanism in SfBt associated with reduced binding of Cry1Fa to BBMV, suggesting that the resistant allele may be affecting expression of a Cry1Fa binding molecule. However, these data remain to be confirmed by further studies for Cry1Fa protein receptors identification. The Cry1Fa resistant population SfBt showed high cross-resistance to Cry1Aa, and low cross-resistance to Cry1Ab and Cry1Ac toxins. The latter proteins are expressed in other transgenic plants used in Brazil, including cotton and soybean, which are also host plants for *S*. *frugiperda* larvae. It remains to be determined if the SfBt population is capable of surviving in other crops expressing Cry1Ab, Cry1Ac or Cry2Ab toxins. Our data indicated no cross-resistance to Cry2A toxin and no susceptibility to Cry2Ab. Due to the low susceptibility of *S*. *frugiperda* to Cry2Aa and Cry2Ab toxins it is expected that GM plants expressing Cry2Ab are not likely to be effective in controlling Cry1Fa resistant insects. Resistance of *S*. *frugiperda* to Cry1Fa corn was previously documented in Puerto Rico [13]. The *S*. *frugiperda* resistant population from Puerto Rico showed a similar cross-resistance pattern to Cry toxins to the SfBt reported here, with low cross-resistance to Cry1Ab and Cry1Ac and no cross-resistance to Cry2Ab toxin [13]. These data suggest that the resistance mechanism of the Puerto Rican and Brazilian populations could be similar, but this remains to be further studied. Other reports also showed cross-resistance among Cry1As and Cry1Fa toxins in other insects resistant to Cry1A toxins such as YHD2 strains from *Heliothis virescens* [34], and NO-QA strain of *Plutella xylostella* [35]. It was recently shown that Cry1Fa resistant population from Puerto Rico was sensitive to DiPel and XenTari Bt formulations [36]. DiPel is based on HD1 *B*. *thuringiensis* var. *kurstaki* strain that expresses the three Cry1Aa, Cry1Ab, Cry1Ac and Cry2Aa toxins while XenTari express Cry1C and Cry1D that are toxic to *S*. *frugiperda* [36]. The high toxicity of DiPel to the Cry1Fa resistant population from Puerto Rico is not clear based on the bioassay data provided here. It is possible that Cry1Aa and Cry2A toxins show different toxicity profiles to both *S*. *frugiperda* populations. Here we show that Cry1Aa is highly toxic to the susceptible strain of SfLab (Table 1). It was previously reported that *S*. *frugiperda* populations from different Latin American regions showed different susceptibility to selected Bt toxins [37] suggesting that the SfLab population may shows important differences in susceptibility to specific Cry toxins when compared with the population from Puerto Rico and this remains to be analyzed.

Competition binding assays have shown that Cry1As and Cry1Fa share binding sites in different Lepidopteran insects such as *H*. *virescens* [38], *Helicoverpa armigera*, *Helicoverpa zea*, *Spodoptera exigua* [39] *Ostrinia nubilalis* and also in *S*. *frugiperda* [33, 40]. Cross-resistance to Cry1Fa and Cry1A toxins could be correlated with sharing binding sites among these toxins. The different cross-resistance levels shown by the different Cry1A toxins suggests that Cry1Fa and Cry1Aa share at least one important binding site involved in toxicity, while Cry1Ab and Cry1Ac share an additional binding site with Cry1Fa that is less important for toxicity. Further work is necessary to demonstrate if binding of Cry1Fa is altered in the resistant strain and to identify the mutated gene in the resistant strain.

As previously reported [33], we found that Cry2A toxins bound to *S*. *frugiperda* BBMV, but we showed here that these toxins showed low toxicity to SfLab or SfBt and did not compete the binding of Cry1Fa to SfLab BBMV. These data indicate that these toxins would not be an alternative to control Cry1Fa resistant populations. Other examples showing lack of correlation between Cry toxin binding to BBMV and insect susceptibility were previously reported [41, 42]. Also toxicity data of non-active mutant toxins that were affected in pore formation but still able to bind to BBMV as the wild type toxin support the idea that binding of Cry toxins to BBMV is necessary but not sufficient to kill the larvae [43–46].

The SfBt population showed a significant cross resistant ratio of 3.1 fold to Cry1AbMod and 5.6 fold to Cry1AcMod toxins (Table 1). It is important to mention that this resistant ratio is similar with native Cry1Ab and Cry1Ac toxins (Table 1). These data suggest that Cry1AMod, Cry1Ab, Cry1Ac and Cry1F share at least one binding site that is not that important for Cry1Ab, Cry1Ac and Cry1AMod toxicity. Nevertheless, Cry1AbMod and Cry1AcMod toxins showed a significant increase in potency to both susceptible and resistant populations (Table 2). Table 1 shows that the LC_50_ value of Cry1AMod toxins to SfBt is comparable to the LC_50_ value of Cry1Fa to SfLab population. The increase in potency ratio is higher than the resistance ratio of both Cry1AMod toxins indicating that even though that SfBt show cross resistance to Cry1AMod, these Cry1AMod toxins are effective in countering resistance of *S*. *frugiperda* to Cry1Fa.

In the model of the mechanism of action of Cry1A toxins, it was proposed that binding to a membrane bound cadherin receptor facilitates removal of the N-terminal region of the toxin including helix alpha-1 and part of alpha-2 resulting in oligomerization of the toxin [3]. Cry1AMod toxins lack this N-terminal region and are able to oligomerize in the absence of cadherin receptor [19] and is proposed that this oligomer requires to bind to GPI anchored receptors and insert into the membrane to kill the larvae [19, 31]. Cry1AMod toxins were shown to counter resistance linked to cadherin mutations and also resistance linked to other resistant mechanisms in different lepidopteran species [19, 20]. Thus, the high insecticidal activity of Cry1AMod toxins against the SfBt and SfLab populations that is even higher than the Cry1Fa toxin (Table 1) suggests that oligomerization is an important step in the mechanism of action of Cry toxins in this insect since a mutant toxin that is able to oligomerize in the absence of protein receptors is highly toxic to *S*. *frugiperda* larvae (Table 1). In this order of ideas it is possible to hypothesize that the resistant allele in SfBt may be affecting a receptor molecule involved in Cry1Fa oligomerization. Further work will determine if resistance is linked to mutations affecting an *S*. *frugiperda* molecule involved in Cy1F toxin binding and oligomerization.

The analysis of resistance mechanisms and the effectiveness of Cry1AMod toxins in other populations of *S*. *frugiperda* from other regions of Brazil should be determined in the future. The data presented here showed the rapid evolution of insect resistance to Bt crops in Brazil and is a warning that in the future a proper use/management of Cry toxins would be needed. Also, we showed that the use of Cry1AMod toxins could provide alternative choices to target *S*. *frugiperda* and possibly manage resistance to Cry1F.
